# Machine Learning-Driven Design and Experimental Validation of a Highly Miniaturized Dual-Band MIMO Antenna for Sub-6 GHz Applications

**DOI:** 10.3390/s26154687

**Published:** 2026-07-23

**Authors:** Ahmet Turgut, Begum Korunur Engiz, Cetin Kurnaz, Muhammet Riza Karadavut

**Affiliations:** 1 Department of Electrical and Electronics Engineering, Faculty of Engineering, Ondokuz Mayis University, 55139 Samsun, Türkiye; 2 3DR Elektronik Sanayi Ticaret, Samsun Technopark, Aksu District, Yurt Street, No. 165, Atakum, 55270 Samsun, Türkiye

**Keywords:** active learning, antenna miniaturization, artificial intelligence, deep surrogate modeling, defected ground structure, MIMO antenna, mutual coupling, sub-6 GHz

## Abstract

The rapid expansion of sub-6 GHz 5G and Internet of Things (IoT) networks demands highly miniaturized Multiple-Input Multiple-Output (MIMO) antennas. However, balancing extreme physical compactness with rigorous inter-port isolation introduces severe computational bottlenecks for conventional optimization algorithms. To overcome these multidimensional challenges, this paper proposes a novel Deep Surrogate Active Learning framework for the autonomous design and empirical validation of an ultra-compact dual-band MIMO antenna. By using a surrogate-assisted closed-loop strategy to reduce reliance on repeated full-wave evaluations, the methodology combined a custom-penalized Deep Neural Network with dynamic boundary reduction. After training the initial surrogate model with 440 valid full-wave responses obtained from the offline design-of-experiments (DOE) stage, the best CST-validated candidate was identified at the 83rd active learning cycle. The optimized nested-loop geometry, incorporating a partial defected ground structure (DGS), occupies an extremely confined footprint of only 1634 mm^2^ on a Rogers RO4350B substrate (Rogers Corporation, Chandler, AZ, USA). The selected geometry provided simulated −10 dB impedance bands of 3.35–3.88 GHz and 4.34–5.05 GHz, while the complete two-port model maintained inter-port isolation better than 13.8 dB and 14.9 dB across the lower and upper target passbands, respectively. Measurements of the fabricated prototype showed the intended dual-band behavior, a maximum measured gain of 4.54 dBi, and total radiation efficiencies of approximately 51–63% across both ports at the evaluated frequencies. The simulated Envelope Correlation Coefficient (ECC) remained below 0.035 across the target passbands, supporting the suitability of the compact geometry for the investigated sub-6 GHz MIMO bands.

## 1. Introduction

The rapid deployment of sub-6 GHz communication networks and the widespread integration of Internet of Things (IoT) devices have fundamentally reshaped the hardware requirements for modern wireless systems [1,2,3]. To support the escalating demand for high data throughput, expanded channel capacity, and reliable link quality in these advanced networks, Multiple-Input Multiple-Output (MIMO) technology has become an indispensable engineering standard [4,5]. However, as the physical dimensions of mobile terminals, smart sensors, and portable wireless platforms continue to shrink, hardware designers face severe spatial constraints. Consequently, the development of highly miniaturized, multi-band MIMO antenna systems that can seamlessly integrate into confined spaces without sacrificing operational efficiency has emerged as a primary challenge in contemporary applied electromagnetics [6,7,8].

While reducing the physical footprint of an antenna is practically desirable, extreme miniaturization inherently introduces severe electromagnetic complexities within a MIMO configuration [9,10]. As multiple radiating elements are placed in close proximity to satisfy spatial limitations, intense electromagnetic interactions occur between the ports, leading to a phenomenon known as mutual coupling [11,12]. High mutual coupling drastically degrades port isolation, alters radiation patterns, and lowers the overall radiation efficiency of the system. Therefore, achieving strict multi-band resonance while simultaneously ensuring high inter-port isolation (e.g., >15 dB) is a formidable task. Furthermore, for a MIMO system to be viable in real-world scattering environments, it must strictly satisfy stringent spatial diversity metrics, including an Envelope Correlation Coefficient (ECC) of less than 0.05, a Total Active Reflection Coefficient (TARC) below −10 dB, and minimal Channel Capacity Loss (CCL) [13,14,15]. Balancing extreme physical compactness with these multi-objective electromagnetic constraints defines an intricate multidimensional design problem [16].

Navigating this complex and nonlinear design space, microwave engineers have traditionally relied on manual parametric sweeps or classical direct search optimization techniques [17]. When manual trial-and-error methods become insufficient for handling strict multi-objective constraints, heuristic global optimizers, such as Genetic Algorithms (GAs), or local search frameworks like the Trust Region Framework (TRF) and the Nelder–Mead Simplex Algorithm (NMSA), are widely employed [18,19]. Although mathematically well established, these conventional algorithms suffer from a profound computational bottleneck known as the curse of dimensionality. To converge on an acceptable optimal geometry, they inherently require a continuous, dense sequence of iterative full-wave electromagnetic solver evaluations [20]. Because each iterative step demands a computationally expensive 3D electromagnetic simulation, executing hundreds or thousands of solver runs incurs a heavy computational cost. This brute-force approach results in unacceptable time consumption and makes traditional optimization techniques inefficient for the rapid prototyping of multi-band, miniaturized MIMO structures [21,22].

Addressing the significant computational burden imposed by traditional iterative solvers, the recent literature has witnessed a paradigm shift toward the integration of artificial intelligence (AI) and machine learning (ML) techniques into RF hardware design [23,24]. Data-driven methodologies, such as Deep Neural Networks (DNNs) and surrogate modeling, have been proposed to bypass conventional parametric sweeps by predicting electromagnetic responses almost instantaneously after an initial training phase [25,26,27,28,29]. Recent empirical analyses have shown that deep learning-based surrogate frameworks can accelerate EM predictions by multiple orders of magnitude without compromising simulation accuracy, effectively outperforming classical algorithms in complex antenna optimizations. Building on these advancements, several studies have utilized various optimization frameworks to develop sub-6 GHz and wideband MIMO configurations [30,31,32,33,34]. Furthermore, state-of-the-art generative deep learning architectures have begun to autonomously orchestrate the structural topology of complex multi-port MIMO arrays to inherently achieve high isolation [35]. While these AI-assisted methods demonstrate remarkable mathematical efficiency, a critical gap remains in the current publishing landscape. A vast majority of contemporary machine learning-based antenna optimizations are strictly simulation-only studies. They frequently rely on theoretically optimized geometries generated entirely in silico, failing to account for real-world fabrication tolerances, substrate dielectric variations, and connector parasitics [36,37]. Consequently, without physical fabrication and empirical validation, such as anechoic chamber measurements, the practical viability and true radiation performance of these mathematically generated MIMO structures remain unproven under real-world conditions [38,39]. Therefore, there is a growing scientific consensus in contemporary applied electromagnetics that autonomous data-driven optimization must be strictly coupled with rigorous experimental hardware validation to assess physical reliability [40].

To bridge the critical gap between theoretical computational efficiency and empirical hardware reliability, this paper proposes the autonomous design, optimization, and comprehensive experimental validation of an ultra-compact dual-band MIMO antenna for sub-6 GHz applications. Methodologically, the conventional parametric sweep and heuristic solver bottlenecks are effectively mitigated through the introduction of a novel Deep Surrogate Active Learning framework. This methodology uses Latin Hypercube Sampling (LHS) to generate the initial offline candidate set and then refines the selected geometry through a closed-loop active learning pipeline with dynamic boundary reduction. For fair benchmarking, the AI framework and the conventional CST optimizers were evaluated under the same predefined solver evaluation budget. In the AI workflow, 500 LHS candidate geometries were generated within this budget, while one baseline geometry was evaluated separately as a common reference. After unsuccessful or interrupted CST runs were excluded, 440 valid full-wave responses, including the baseline response, were retained for the initial surrogate model training. Starting from this valid database, the best CST-validated candidate was identified at the 83rd active learning cycle and was subsequently compared with the CST built-in GA, TRF, and NMSA results. Structurally, the selected geometry occupies a compact footprint of 1634 mm^2^ (25.89 mm × 63.11 mm). In the complete two-port model, inter-port isolation was better than 13.8 dB across the lower target passband and 14.9 dB across the upper target passband, while the simulated ECC remained below 0.035 across the target passbands and the TARC and CCL satisfied their respective acceptance limits within the effective core MIMO sub-bands. The geometry was subsequently fabricated on an RO4350B substrate, and its scattering parameters, normalized two-dimensional radiation patterns, peak gain, directivity, and total radiation efficiency were experimentally evaluated using calibrated measurement systems. The present implementation and validation are limited to the parameterized nested loop dual-band sub-6 GHz topology considered in this study; application of the framework to other antenna architectures or frequency ranges would require redefinition of the design variables and model retraining using topology-specific full-wave data.

The remainder of this article is organized as follows: Section 2 details the geometric layout of the proposed miniaturized MIMO antenna and describes the mathematical architecture of the Deep Surrogate Active Learning framework, together with a comprehensive optimization benchmark. Section 3 presents the simulated electromagnetic performance of the AI-optimized structure, specifically analyzing impedance matching, mutual coupling suppression, and spatial diversity metrics. Section 4 provides the physical prototype details, describing the fabrication process and presenting the physical measurements conducted within the anechoic chamber. Section 5 presents a comprehensive state-of-the-art comparison, benchmarking the proposed antenna against the recent literature. Finally, Section 6 concludes the paper by summarizing the major contributions.

## 2. Antenna Geometry and AI-Driven Methodology

Designing a highly miniaturized dual-band MIMO antenna for sub-6 GHz applications requires navigating a highly nonlinear multidimensional design space, where the objectives of extreme physical compactness and rigorous inter-port isolation strictly conflict. Conventional direct search and heuristic optimization methodologies generally struggle to balance these multi-objective constraints without incurring an immense computational burden and frequently become trapped in local minima [41]. To overcome this structural challenge, this study completely decouples the physical design phase from traditional trial-and-error workflows by deploying an autonomous machine learning architecture. This section first delineates the physical topology of the proposed nested loop MIMO structure, focusing on its defected ground isolation mechanics and geometric parameterization. Following the physical definitions, the operational methodology of the proposed Deep Surrogate Active Learning framework—which uses a data-driven surrogate to reduce reliance on repeated full-wave evaluations while retaining CST validation within the closed loop—is detailed, together with a controlled algorithmic benchmark conducted under a common maximum budget of 800 candidate evaluations.

### 2.1. Antenna Layout and Design Parameters

The geometric configuration of the proposed highly miniaturized dual-band MIMO antenna is meticulously designed to operate seamlessly within the sub-6 GHz spectrum. To minimize dielectric losses at high frequencies and ensure structural stability, the antenna is modeled and fabricated on a low-loss Rogers RO4350B substrate characterized by a relative permittivity (relative_permittivity) of 3.48, a loss tangent (tan_delta) of 0.0037, and a standard thickness (substrate_height) of 0.762 mm. Driven by the stringent spatial constraints of modern portable wireless devices, the complete physical footprint of the MIMO configuration is extremely confined. The total vertical length of the substrate (substrate_length) is determined by the sum of the ground plane length (groundplane_length) and the dielectric extension length (dielectric_extension_length), equating to precisely 25.89 mm. The total horizontal width is fixed at 63.11 mm, yielding an overall compact physical area of 1634 mm^2^.

**Figure 1:**
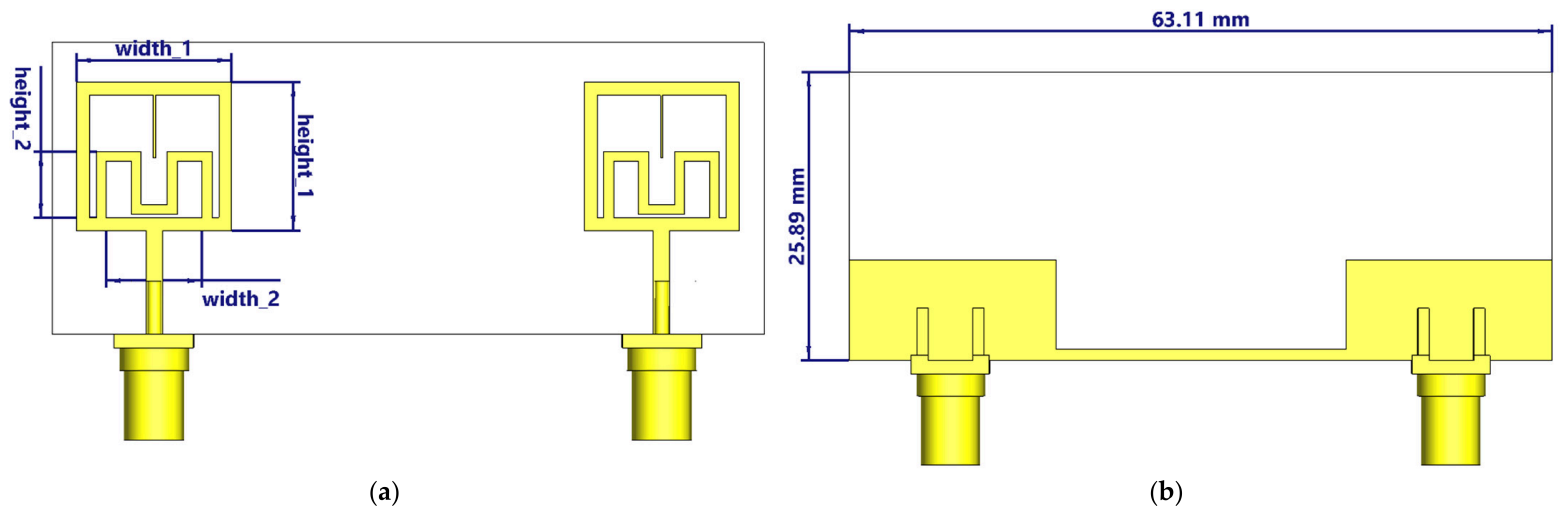
Geometrical layout of the proposed miniaturized dual-band MIMO antenna: (**a**) top view; (**b**) bottom view.

As illustrated in Figure 1, the primary radiating structure, located on the top layer, consists of two symmetrically positioned resonating elements separated by an edge-to-edge inter-element distance (wd) of 26.83 mm. To achieve dual-band resonance without physically enlarging the radiating area, a shared feed nested loop topology was engineered. Each radiating element features an outer rectangular loop that governs the fundamental lower-frequency band (3.55 GHz). Nested within this outer perimeter is an inner meandered loop structure. By incorporating an M-shaped meander section with a predefined physical length (length_1) of 5.57 mm and an inner gap distance (length_2) of 2.20 mm, the electrical current path is significantly extended within a constrained physical boundary, thereby triggering the higher-frequency resonance (4.70 GHz). Furthermore, a narrow vertical tuning stub (monopole element) with a precise line width (line_width_3) of 0.015 mm and height (height_3) of 5.55 mm is extruded inwards from the upper-center of the outer loop. This localized ultra-thin metallic trace functions as a crucial capacitive tuning element to critically match the input impedance for the upper band. Each element is independently excited via a 50 Ω microstrip feedline with a calculated width (feed_line_width) of 1.47 mm and a length (feed_line_length) of 9.20 mm. The trace widths of the outer loop (line_width_1) and the inner loop (line_width_2) are fixed at 1.14 mm and 0.88 mm, respectively.

To combat the intense mutual coupling conventionally associated with such compact spatial arrangements, a defected ground structure (DGS) is deliberately integrated into the bottom layer. Instead of utilizing a continuous ground plane, a partial ground configuration is employed, featuring a substantial rectangular defect between the two individual ground sections. Each partial ground section has a length (groundplane_length) of 9.00 mm and a width (groundplane_width) of 18.14 mm, separated by a defined ground plane gap width (gw) of 0.40 mm. This strategically placed clearance disrupts the ground current path, thereby suppressing surface wave propagation and reducing near-field coupling between the antenna elements. Consequently, it acts as a self-decoupling mechanism that maintains inter-port isolation better than 13.8 dB across the lower target passband and 14.9 dB across the upper target passband without requiring additional parasitic components. This structural simplicity inherently aligns with recent deep learning optimization strategies aimed at natively maximizing inter-port isolation within spatially constrained printed circuits, entirely circumventing the need for external complex decoupling layers [42,43].

For the purpose of scientific transparency and to rigorously define the state space for the subsequent machine learning pipeline, the physical dimensions are bifurcated into static analytical constants and dynamic optimization variables. While the fundamental feeding, meandering, and ground-decoupling parameters were kept constant, the four geometric variables defining the inner and outer loops—namely height_1, height_2, width_1, and width_2—were used as the search variables of the proposed surrogate-assisted optimization framework. The complete list of geometrical parameters, strictly adhering to the exact simulation nomenclature, is comprehensively summarized in Table 1.

### 2.2. Proposed Deep Surrogate Active Learning Framework

Conventional population-based optimization algorithms that natively operate within commercial 3D solvers typically execute sequential search routines. Operating without prior topographical knowledge of the design space, these direct search algorithms frequently become trapped in local minima when confronted with highly nonlinear multi-objective antenna geometries. To overcome this computational bottleneck, a Closed-Loop Deep Surrogate Active Learning framework is proposed.

**Figure 2:**
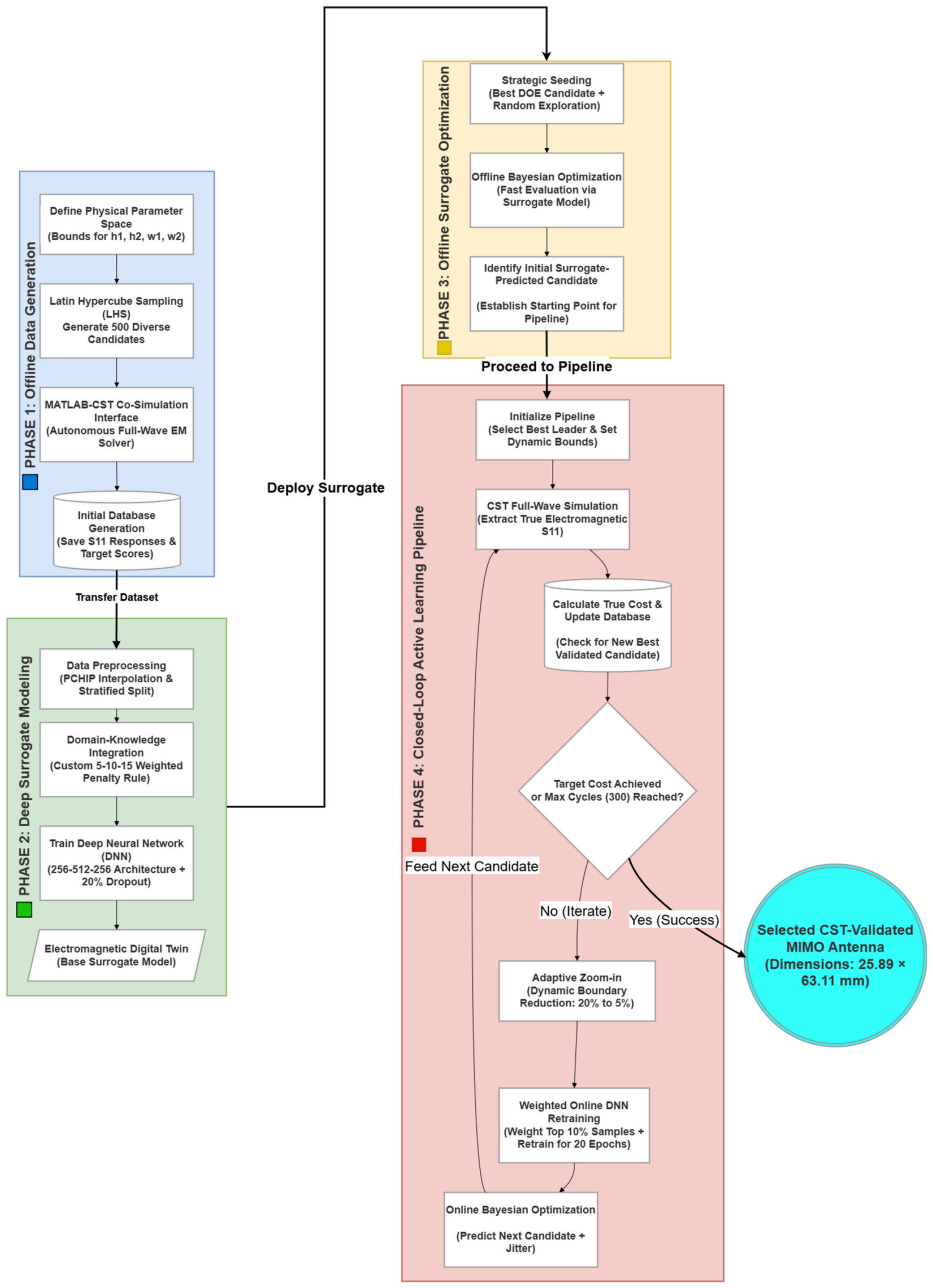
Flowchart of the proposed deep surrogate active learning framework.

To ensure strict scientific reproducibility, all electromagnetic (EM) simulations and machine learning training pipelines were accelerated utilizing an NVIDIA RTX A4000 GPU (16 GB) on a high-performance workstation equipped with an Intel Core i7-7700K CPU (4.20 GHz) (Santa Clara, CA, USA) and 32 GB of RAM. The full-wave EM evaluations were executed using CST Studio Suite 2025, while the deep learning and autonomous optimization pipelines were scripted in MATLAB R2023a via a custom co-simulation Application Programming Interface (API). As depicted in the operational flowchart in Figure 2, the proposed framework structurally decouples the EM solving process from the optimization search and operates through four interconnected phases:

Phase 1—Offline Data Generation: To map the multidimensional design space without an exhaustive grid sweep, the four dynamic geometric variables (height_1, height_2, width_1, and width_2) were sampled using Latin Hypercube Sampling (LHS) within the predefined bounds [44]. A total of 500 LHS candidate geometries were generated within the benchmarking budget, while one baseline geometry was evaluated separately as a reference. All candidates were autonomously submitted to CST Studio Suite 2025 through the MATLAB–CST co-simulation interface implemented in MATLAB R2023a. After excluding unsuccessful or interrupted solver runs, 440 valid full-wave responses, including the baseline response, were retained for the initial surrogate model training.

Phase 2—Deep Surrogate Modeling and Domain Knowledge Integration: The raw frequency responses were preprocessed using Piecewise Cubic Hermite Interpolating Polynomial (PCHIP) interpolation to ensure consistent frequency vectors, followed by a stratified data split for robust training and validation. To construct the surrogate model, a Deep Neural Network (DNN) was engineered with a 256-512-256 fully connected layer architecture. To reduce overfitting and improve predictive generalization within the sampled four-dimensional design space, Leaky ReLU activation functions and a 20% Dropout layer were incorporated [45].

Domain knowledge was incorporated through frequency-dependent output weighting during surrogate training and a problem-specific cost function. The cost function, Cost(x), was formulated to prioritize dual-band impedance matching, out-of-band rejection, and band-edge targets:

(1) $$Cost\left(x\right)=\sum_{f\in F_{pass}}15\max\left(0,S_{11}\left(x,f\right)+25)+\sum_{f\in F_{stop}}5\max\left(0,-10-S_{11}\left(x,f\right))+\sum_{f\in F_{corner}}10\left|S_{11}\left(x,f\right)+10\right|\right)\right)$$

where x represents the geometric input vector, and S_11_(x,f) is the reflection coefficient magnitude in dB at a given frequency f. The algorithm assigns a higher penalty weight of 15 for the operational target bands (F_pass_: 3.3–3.8 GHz and 4.4–5.0 GHz) to prioritize a deep resonance of ≤−25 dB. Out-of-band rejection regions (F_stop_: <3.3 GHz, 3.8–4.4 GHz, and >5.0 GHz) are assigned a weight of 5 to maintain the signal above −10 dB, ensuring high frequency selectivity. Finally, critical corner frequencies (F_corner_: 3.3, 3.8, 4.4, and 5.0 GHz) are anchored with a weight of 10 to encourage sharp band-edge transitions.

Phase 3—Offline Surrogate Optimization: Once the DNN had been trained as a computationally efficient surrogate of the full-wave model, an offline Bayesian optimization routine was performed using the expected-improvement-plus acquisition function [46]. The search comprised 150 surrogate objective evaluations and was initialized using the best candidate from the valid design-of-experiments (DOE) database together with two random exploration points. Because this stage operated exclusively on the DNN predictions rather than on new full-wave evaluations, its output was treated as an initial surrogate-predicted candidate for the subsequent closed-loop active learning stage, rather than as a final global optimum.

Phase 4—Closed-Loop Active Learning Pipeline: To correct prediction deviations between the surrogate model and the full-wave solver, a closed-loop active learning pipeline was configured for 300 online cycles. Together with the 500 LHS trials in the offline DOE stage, this provided the proposed framework with the same predefined 800-evaluation search budget used for benchmarking the conventional CST optimizers; the baseline geometry was evaluated separately as a reference. The online stage started from the database of 440 valid full-wave responses. At each cycle, the candidate proposed by the surrogate optimizer was evaluated in CST, and the objective cost was recalculated directly from the resulting full-wave S_11_ response before the database was updated.

Two adaptive mechanisms were employed during this process. First, the geometric search interval was progressively reduced from ±20% to ±5% around the current best validated candidate. Second, after each successful CST evaluation, the DNN was retrained for 20 epochs using the complete historical database with additional weighting of the best-performing 10% of the samples. A 25-evaluation Bayesian search was then used to propose the next candidate. Of the 300 attempted online cycles, 295 produced valid stored CST responses, expanding the validated database from 440 to 735 entries. The best CST-validated geometry corresponded to RunID 523 and was identified at the 83rd active learning cycle. The predefined campaign was subsequently continued to complete the benchmarking protocol.

### 2.3. Optimization Performance and Computational Efficiency

To evaluate the computational efficiency of the proposed Deep Surrogate Active Learning framework, a controlled benchmarking study was conducted against the built-in Genetic Algorithm (GA), Trust Region Framework (TRF), and Nelder–Mead Simplex Algorithm (NMSA) available in CST Microwave Studio. All methods used the same four-dimensional geometric search space, parameter bounds, CST antenna model, and target mask definitions. A maximum search budget of 800 candidate evaluations was adopted for the comparison. For the proposed framework, this budget consisted of 500 offline LHS trials and 300 online active learning attempts, while the baseline geometry was evaluated separately as a common reference.

The recorded evaluation counts and the resulting geometric parameters are summarized in Table 2. NMSA terminated after 144 evaluations without satisfying all of the prescribed dual-band target masks. TRF completed 799 recorded evaluations; its best candidate was obtained at Run ID 176, after which no further improvement in the best objective value was observed. GA completed 801 recorded evaluations, consisting of 800 solver runs and one reloaded result, but its selected geometry did not provide acceptable matching in the upper target band. In comparison, the proposed framework identified its best CST-validated geometry at the 83rd active learning cycle, corresponding to Run ID 523 in the final database. Among the compared solutions, this geometry provided the most balanced simultaneous satisfaction of the lower-band, upper-band, and inter-band target requirements.

For computational reproducibility, the number of full-wave CST evaluations was used as the primary cost metric because a continuous historical wall clock log was not retained for the complete campaign. A representative end-to-end CST evaluation time was therefore measured on the same workstation and under the same solver configuration using three independent full-wave runs. The mean evaluation time was 166.06 ± 1.72 s, corresponding to approximately 2.77 min per candidate. On this basis, the 83 active learning evaluations required to reach the best CST-validated candidate correspond to an estimated solver-equivalent time of 3.83 h, while the 523 valid evaluations comprising the 440-response initial database and the subsequent 83 active evaluations correspond to approximately 24.13 h. The measured MATLAB-side overhead was comparatively small: one representative 2000-epoch DNN training run required 252.23 s, the 150-evaluation offline surrogate Bayesian optimization required 28.59 s, and the online update—comprising weighted data preparation, 20-epoch retraining, and 25 surrogate Bayesian evaluations—required an average of 4.30 s per cycle. These values are reported as representative measurements and solver-equivalent estimates rather than reconstructed historical wall clock logs.

To complement the surrogate model predictions with an interpretable assessment of the design space, a database-level parameter association analysis was conducted using all 735 CST-validated responses. For each response metric, the four geometric parameters were standardized, a multivariable linear regression was fitted, and the absolute standardized coefficients were normalized to sum to 100%. Across the full database, width_1 showed the largest relative association with the objective cost (42.3%), while height_1 had the strongest association with the lower-resonance frequency (52.7%). The upper-resonance frequency was associated primarily with width_1 (55.9%), whereas height_2 showed the largest association with the upper-band resonance depth (48.9%). A local analysis was also performed using the 10% of database samples nearest to the best CST-validated candidate in the normalized four-dimensional parameter space. Within this local neighborhood, the relative association with the objective cost shifted toward height_2 (56.0%). This difference indicates that the parameters associated with global design space variation are not necessarily identical to those governing local refinement near the selected candidate. These results represent database-level associations rather than direct causal explanations based on the internal DNN weights.

**Figure 3:**
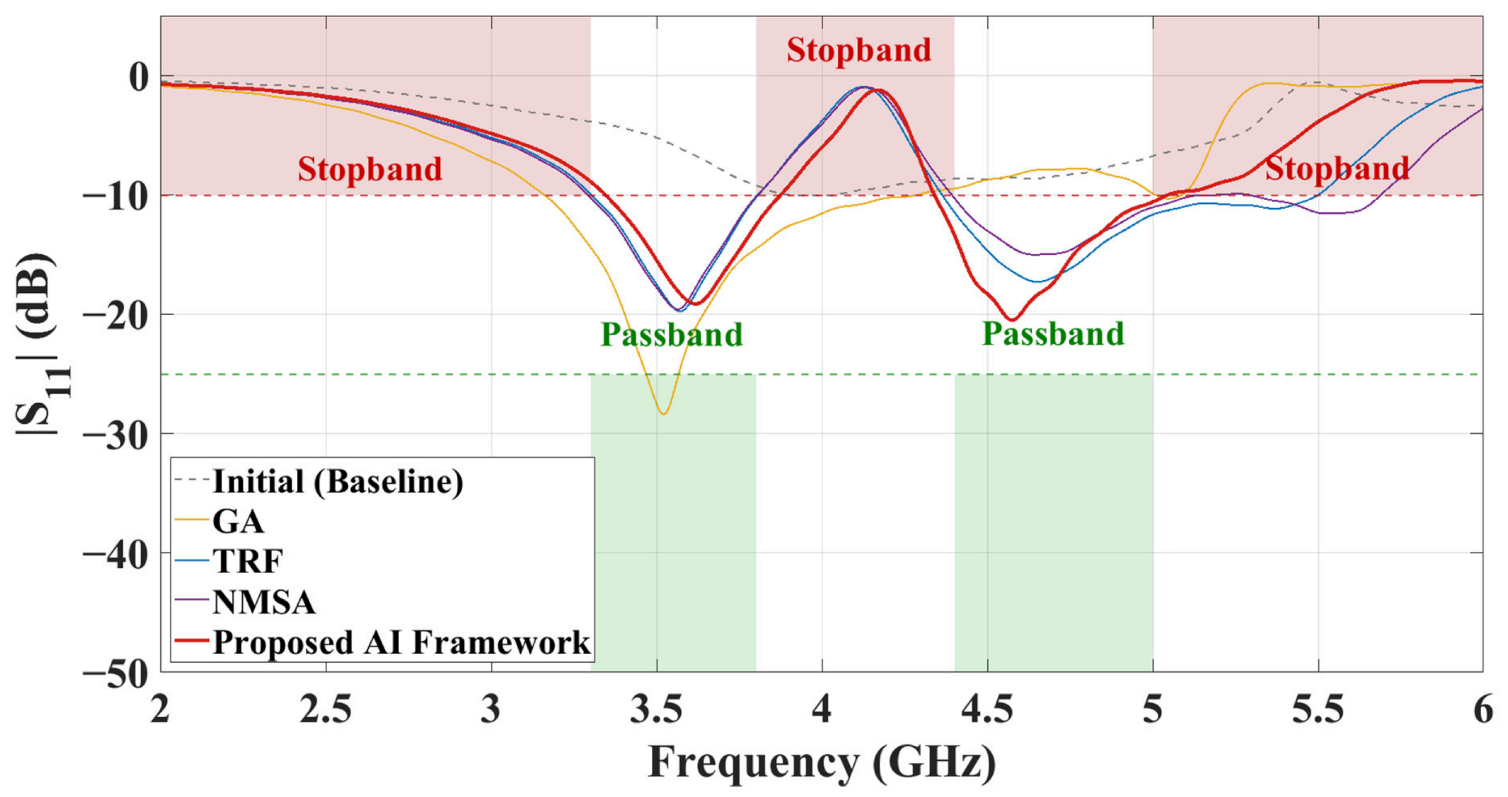
Simulated |S_11_| performance comparison of the initial geometry, conventional CST optimization solvers (GA, TRF, NMSA), and the proposed AI framework, validated against the predefined target masks (passbands in green, stopbands in red).

The electromagnetic consequences of the different optimization outcomes are visible in the simulated reflection coefficient (|S_11_|) responses plotted in Figure 3. The responses were extracted from the exported Touchstone files. Under the penalty formulation defined in Equation (1), the proposed framework produced dual-band resonances at 3.62 GHz (−19.14 dB) and 4.58 GHz (−20.50 dB), with −10 dB impedance bands of 3.35–3.88 GHz and 4.34–5.05 GHz. In the 3.8–4.4 GHz inter-band region, the maximum reflection coefficient remained at −1.23 dB, satisfying the prescribed out-of-band mask.

By comparison, the conventional solvers did not satisfy all of the prescribed target masks. GA produced a deep lower-band resonance of −28.42 dB at 3.52 GHz, but its response in the 4.4–5.0 GHz target region remained around −8.0 dB and did not reach the −10 dB matching criterion. NMSA and TRF produced dual-resonance responses, with upper-band minima of −15.02 dB and −17.30 dB, respectively. Both methods also reached approximately −11.7 dB beyond 5.0 GHz, thereby violating the specified upper-stopband condition of |S_11_| > −10 dB. Overall, within the common evaluation budget, the proposed framework provided the most balanced simultaneous satisfaction of the lower-band, upper-band, and inter-band target requirements. Comparisons with alternative surrogate architectures and multi-objective acquisition strategies were outside the scope of the present benchmark and will be investigated in future work.

## 3. Simulated Electromagnetic and MIMO Performance

While algorithmic efficiency highlights the computational success of the proposed Deep Surrogate Active Learning framework, the practical viability of the generated antenna geometry must be rigorously validated through its physical and electromagnetic characteristics. This section evaluates the simulated full-wave performance of the AI-optimized highly miniaturized MIMO antenna. The analysis is systematically divided into two distinct validation stages. First, the fundamental impedance matching and inter-port isolation mechanics (mutual coupling) are investigated using scattering parameters and surface current distributions. Second, the crucial spatial multiplexing metrics—including the Envelope Correlation Coefficient (ECC), Diversity Gain (DG), Total Active Reflection Coefficient (TARC), and Channel Capacity Loss (CCL)—are evaluated to ensure the antenna satisfies the rigorous spatial diversity standards required for robust sub-6 GHz MIMO operations [47].

### 3.1. Impedance Matching and Mutual Coupling Suppression

**Figure 4:**
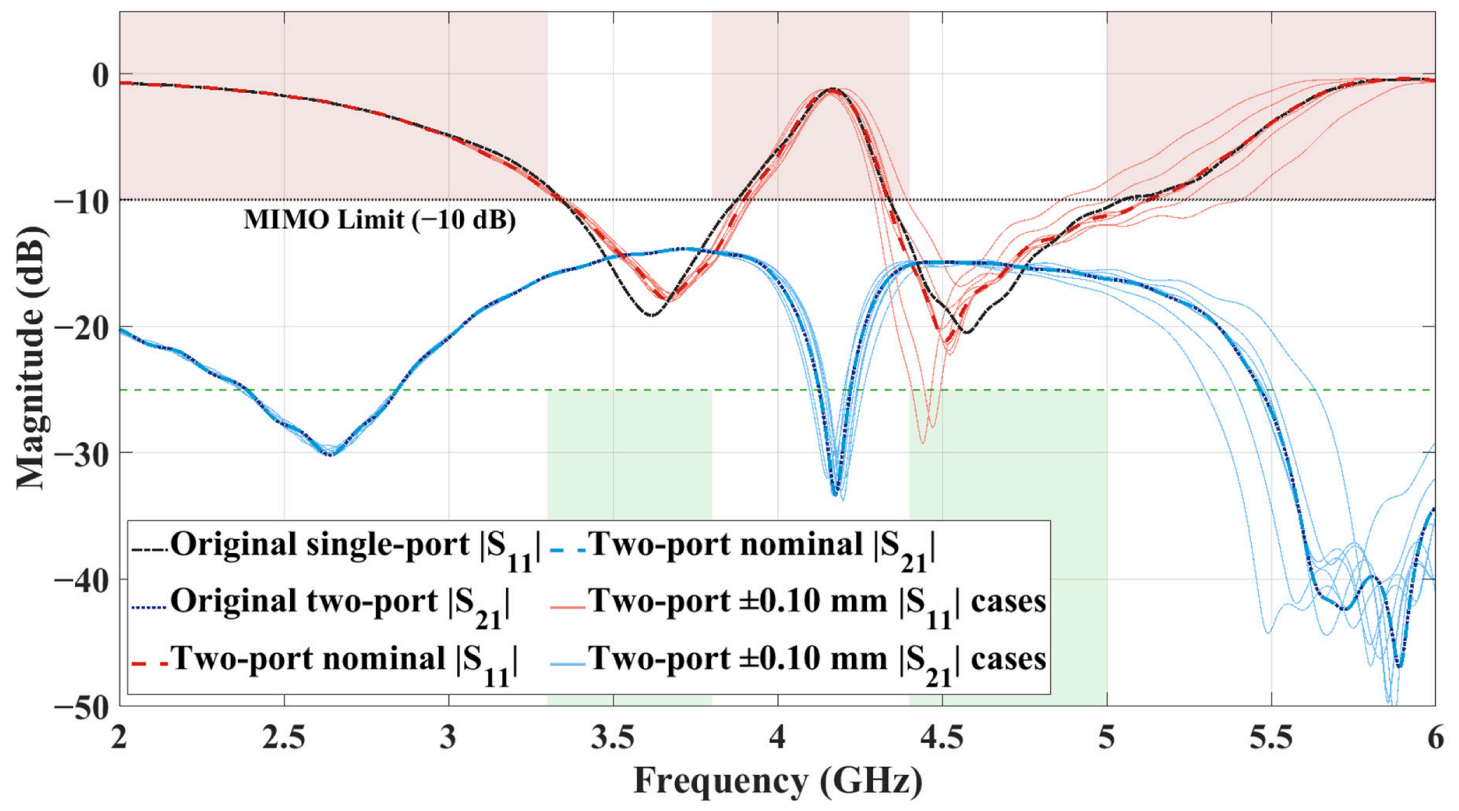
Comparison of the original single-port and complete two-port S-parameter responses with the ±0.10 mm fabrication-tolerance envelope. Black and dark-blue curves denote the original responses, red and light-blue dashed curves denote the nominal two-port responses, and pale-red and pale-blue curves denote the tolerance cases.

The fundamental prerequisites for a reliable MIMO system are maintaining deep impedance matching across the desired frequency bands and ensuring minimal electromagnetic leakage (mutual coupling) between the closely spaced radiating ports. Figure 4 compares the optimization-stage single-port |S_11_| response with the nominal and fabrication tolerance |S_11_| and |S_21_| responses of the complete two-port MIMO model.

The reflection characteristics of the selected CST-validated geometry confirm the intended dual-band response of the predefined shared feed nested loop topology. The outer rectangular loop structure successfully governs the lower operating band, generating an excellent impedance match of −19.14 dB at a resonant frequency of 3.62 GHz. Concurrently, the meandered inner loop, tightly coupled with the capacitive tuning stub, excites the higher operating band, achieving a deep resonance of −20.50 dB at 4.58 GHz. The corresponding −10 dB impedance bands span 3.35–3.88 GHz and 4.34–5.05 GHz, covering several designated sub-6 GHz communication bands. Furthermore, the AI framework successfully constrained the signal response within the inter-band rejection region (3.8–4.4 GHz), keeping the reflection coefficient safely above the −10 dB threshold (peaking at −1.23 dB) to ensure strict frequency selectivity and out-of-band noise rejection.

The resonance frequencies of 3.62 and 4.58 GHz correspond to the single-port model used during the optimization stage. When the second port was included as a matched port in the complete two-port MIMO model, the corresponding resonances shifted to approximately 3.66 and 4.51 GHz, respectively, while the intended dual-band response was preserved. Despite the extremely confined edge-to-edge inter-element spacing of only 26.83 mm, the system demonstrates strong mutual coupling suppression. As depicted by the simulated |S_21_| curve, the inter-port isolation remains better than 13.8 dB over the lower target passband of 3.3–3.8 GHz and better than 14.9 dB over the upper target passband of 4.4–5.0 GHz. This isolation performance was achieved across both target passbands without additional parasitic elements or complex external decoupling networks that would enlarge the antenna dimensions.

To evaluate the local sensitivity of the selected geometry to plausible dimensional deviations, a one-factor-at-a-time fabrication tolerance analysis was conducted. The four AI-optimized dimensions—height_1, height_2, width_1, and width_2—were individually perturbed by ±0.10 mm, while all remaining geometrical parameters, material properties, mesh settings, and solver configurations were kept unchanged. Across the eight perturbed cases, both resonant regions were retained, with maximum resonance frequency shifts of 24 MHz in the lower band and 72 MHz in the upper band relative to the nominal response. The shallowest resonance minima remained below −17.36 dB and −16.83 dB in the lower and upper bands, respectively. The inter-port coupling response was also stable, with the worst-case |S_21_| values remaining at or below −13.87 dB over 3.3–3.8 GHz and −14.76 dB over 4.4–5.0 GHz. The largest upper-band displacement occurred for the −0.10 mm perturbation of height_2, indicating that the upper resonance is comparatively more sensitive to this dimension. Overall, the results demonstrate that the dual-band resonance and isolation characteristics are preserved within the investigated ±0.10 mm local tolerance range, although greater variation is observed at the upper-band edges.

**Figure 5:**
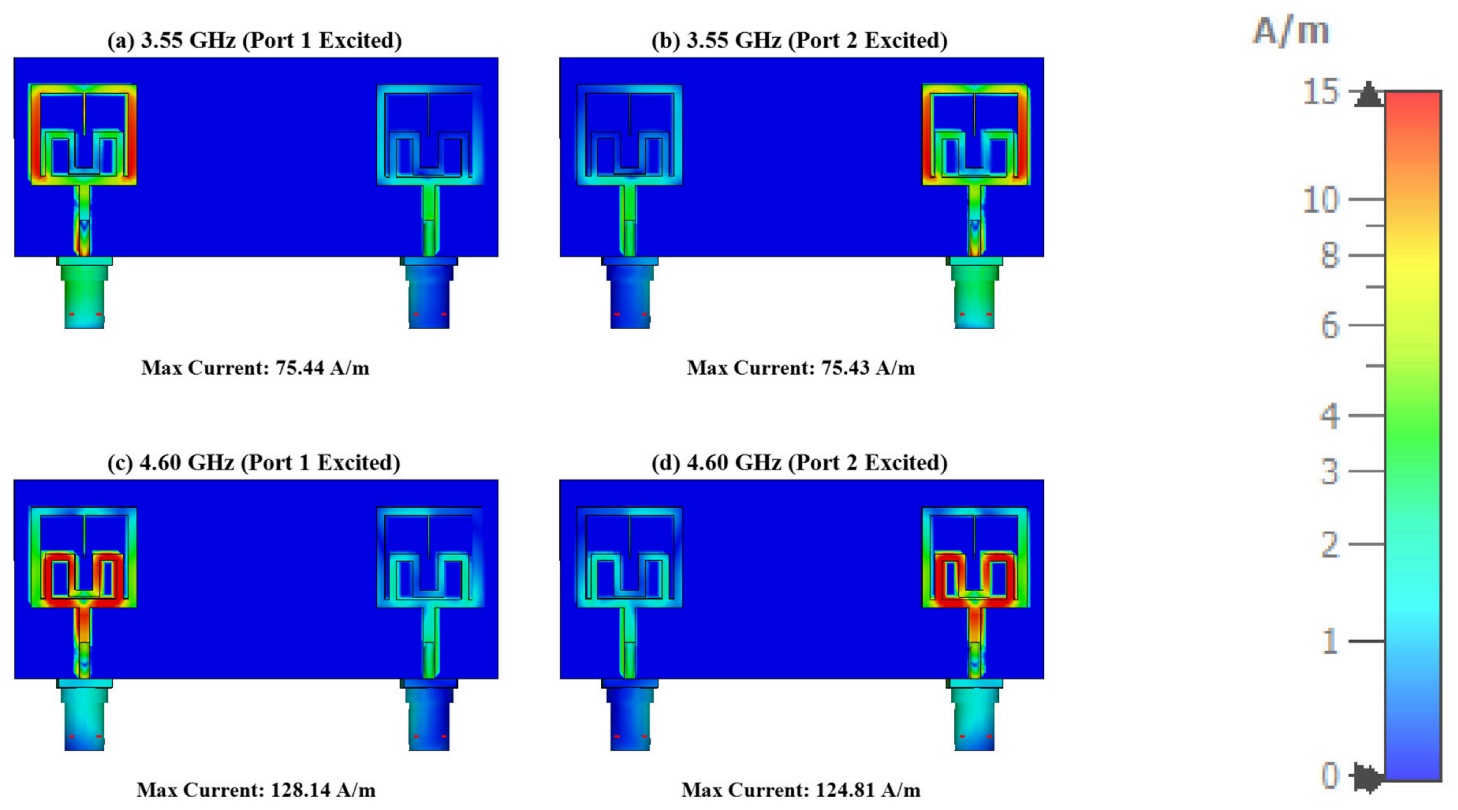
Simulated surface current distributions of the proposed antenna under individual port excitation: (**a**) 3.55 GHz with Port 1 excited; (**b**) 3.55 GHz with Port 2 excited; (**c**) 4.60 GHz with Port 1 excited; (**d**) 4.60 GHz with Port 2 excited.

To examine the current localization and inter-element coupling behavior, simulated surface current distributions were evaluated at the selected operating frequencies of 3.55 and 4.60 GHz. Figure 5 presents the distributions when either Port 1 or Port 2 is independently excited, while the other port is terminated with a 50-ohm matched load. For visual comparison, the displayed color scale was capped at 15 A/m, whereas the annotated values indicate the CST-computed maximum current magnitude for each excitation case.

When Port 1 is excited at 3.55 GHz (Figure 5a), the strongest surface current concentration occurs primarily along the outer rectangular loop of the active element, consistent with its dominant contribution to the lower operating band. The current induced on the non-excited element remains substantially lower than that on the excited element, supporting the limited inter-element coupling indicated by the simulated |S_21_| response. A closely mirrored distribution is observed when Port 2 is excited (Figure 5b), with CST-computed maximum current values of 75.44 A/m and 75.43 A/m for Port 1 and Port 2 excitation, respectively.

At 4.60 GHz (Figure 5c,d), the strongest current regions are concentrated around the inner meandered loop and the adjoining feed region, consistent with the contribution of the nested inner structure to the upper operating band. In both excitation cases, the current induced on the non-excited element remains lower than that on the active element. The CST-computed maximum current values are 128.14 A/m for Port 1 excitation and 124.81 A/m for Port 2 excitation. These distributions support the interpretation of localized upper-band excitation and reduced coupling between the two antenna elements.

### 3.2. Spatial Multiplexing and Channel Metrics

While mutual coupling evaluates the signal leakage between ports, the true spatial multiplexing capability of a MIMO system in a rich multipath scattering environment is determined by its correlation and channel capacity metrics. To comprehensively assess the diversity performance of the AI-optimized antenna, the Envelope Correlation Coefficient (ECC), Diversity Gain (DG), Total Active Reflection Coefficient (TARC), and Channel Capacity Loss (CCL) were mathematically extracted from the simulated complex S-parameters [48].

**Figure 6:**
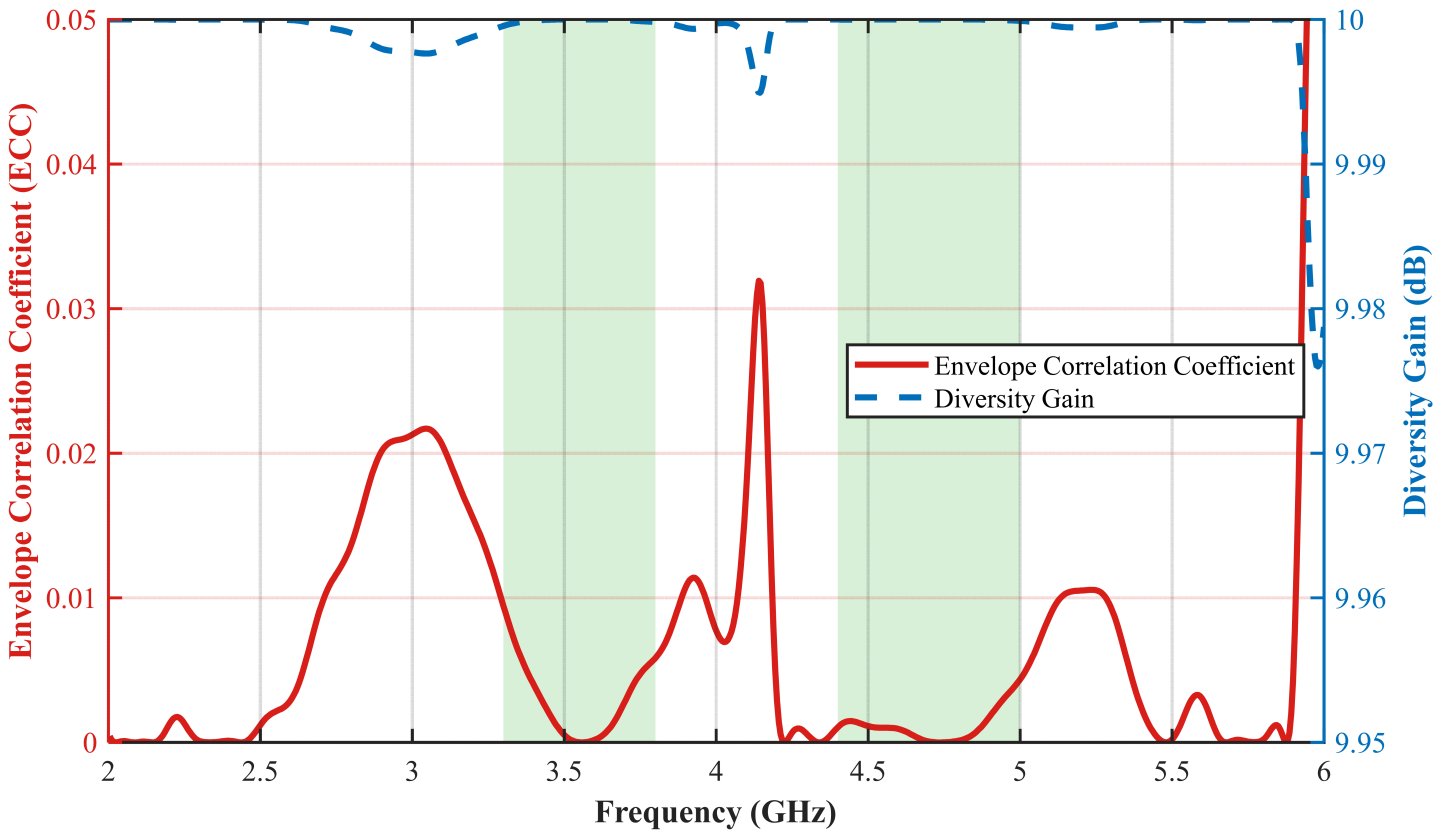
Simulated Envelope Correlation Coefficient (ECC) and Diversity Gain (DG) of the AI-optimized MIMO antenna. The red solid curve represents ECC on the left axis, while the blue dashed curve represents DG on the right axis.

The ECC evaluates the degree of correlation between the radiation patterns of the respective antenna elements. For modern wireless applications, an ECC of less than 0.05 is rigorously demanded to ensure that multiple data streams can be recovered without interference. Correspondingly, the DG measures the effective improvement in the signal-to-noise ratio during MIMO operations and should theoretically approach the maximum of 10 dB. As illustrated in the dual-axis plot in Figure 6, the simulated ECC for the proposed antenna remains exceptionally low. Across the target operating bands, the ECC stays well below 0.035, while the DG consistently fluctuates above 9.95 dB. These values indicate that the shared feed nested loop configuration and the defected ground structure successfully yield highly uncorrelated spatial channels.

**Figure 7:**
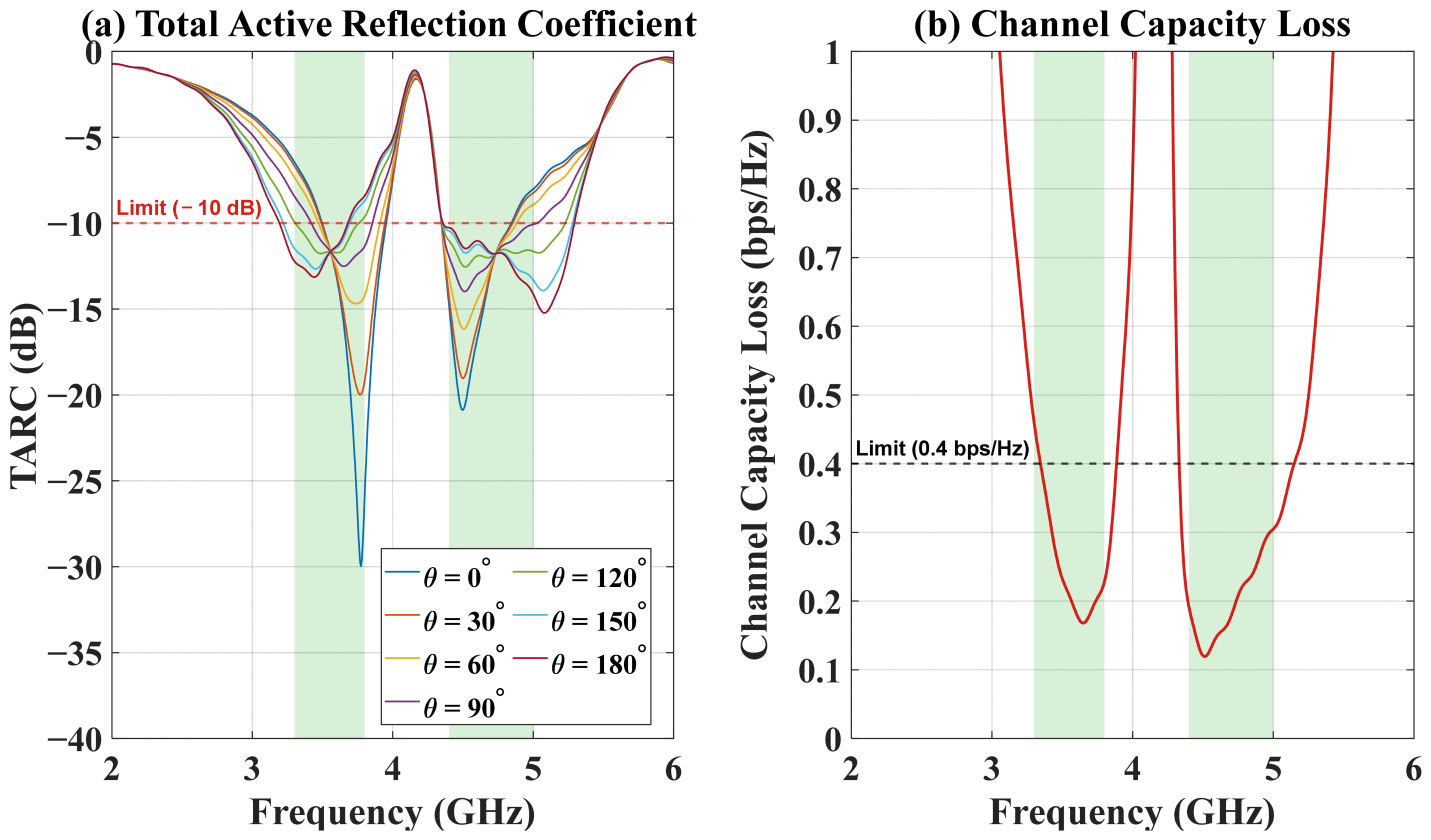
Simulated active spatial metrics: (**a**) Total Active Reflection Coefficient (TARC), where the colored solid curves represent phase differences from θ = 0° to 180° and the red dashed line indicates the −10 dB limit; (**b**) Channel Capacity Loss (CCL), where the red solid curve represents CCL and the black dashed line indicates the 0.4 bps/Hz limit. The green shaded regions indicate the target passbands.

Beyond passive correlation, a MIMO system must be resilient to simultaneous port excitations with arbitrary phase differences. TARC is an active performance metric that recalculates the overall reflection coefficient by accounting for simultaneous excitations under varying input phase angles (θ). Similarly, the CCL evaluates the maximum potential data transmission rate loss caused by the residual correlation between the elements, with a strict acceptable threshold of ≤0.4 bps/Hz. Figure 7 illustrates the simulated TARC for sweeping phase differences from 0° to 180° alongside the CCL performance.

As theoretically anticipated in highly miniaturized multi-band structures, active MIMO metrics exhibit increased sensitivity near the absolute edges of the passive impedance bandwidths. While the passive reflection coefficient spans the entire predefined target areas, the worst-case phase combinations (e.g., θ = 150° and 180°) in the TARC evaluation vectorially compound the mutual coupling, causing the active reflection to slightly exceed the −10 dB limit precisely at the band boundaries. To accurately represent the operational capability under the harshest active conditions, the effective core MIMO sub-bands are strictly defined as 3.45–3.75 GHz and 4.45–4.90 GHz. Within these primary resonant regions, the antenna maintains extraordinary resilience. The TARC strictly drops below −10 dB regardless of the phase angle variations, ensuring highly stable active impedance matching. Concurrently, the CCL drops significantly below the 0.4 bps/Hz threshold, reaching minimums near 0.15 bps/Hz in both core sub-bands. These results indicate that the proposed geometry maintains acceptable active matching and channel capacity performance within the defined core MIMO sub-bands.

## 4. Fabrication and Experimental Validation

While the simulated electromagnetic metrics confirm the theoretical robustness of the AI-optimized MIMO antenna, computational models natively operate under mathematically idealized boundary conditions. In practical applications, physical factors such as minor substrate dielectric variations, chemical etching tolerances, and parasitic near-field coupling introduced by metallic connectors inevitably perturb the actual operational characteristics of an antenna [36]. To evaluate the critical gap between in silico optimization and practical hardware reliability, the proposed AI-generated geometry was physically manufactured and subjected to a rigorous empirical evaluation.

The experimental validation protocol was systematically executed in two sequential stages to evaluate both the circuit-level network parameters and the spatial radiation properties. Initially, the passive scattering parameters, encompassing both impedance matching and inter-port mutual coupling, were measured using a calibrated Anritsu MS2037C VNA Master vector network analyzer (Atsugi, Japan). Subsequently, to validate the spatial radiation characteristics—including two-dimensional radiation patterns, peak gain, directivity, and total radiation efficiency—the prototype was evaluated within a professional Spherical Near-Field Measurement System (SNFMS). These comprehensive far-field measurements were conducted at the TUBITAK BILGEM Antenna Test and Research Center (ATAM), strictly adhering to standard antenna test procedures.

### 4.1. Prototype Details and S-Parameter Validation

**Figure 8:**
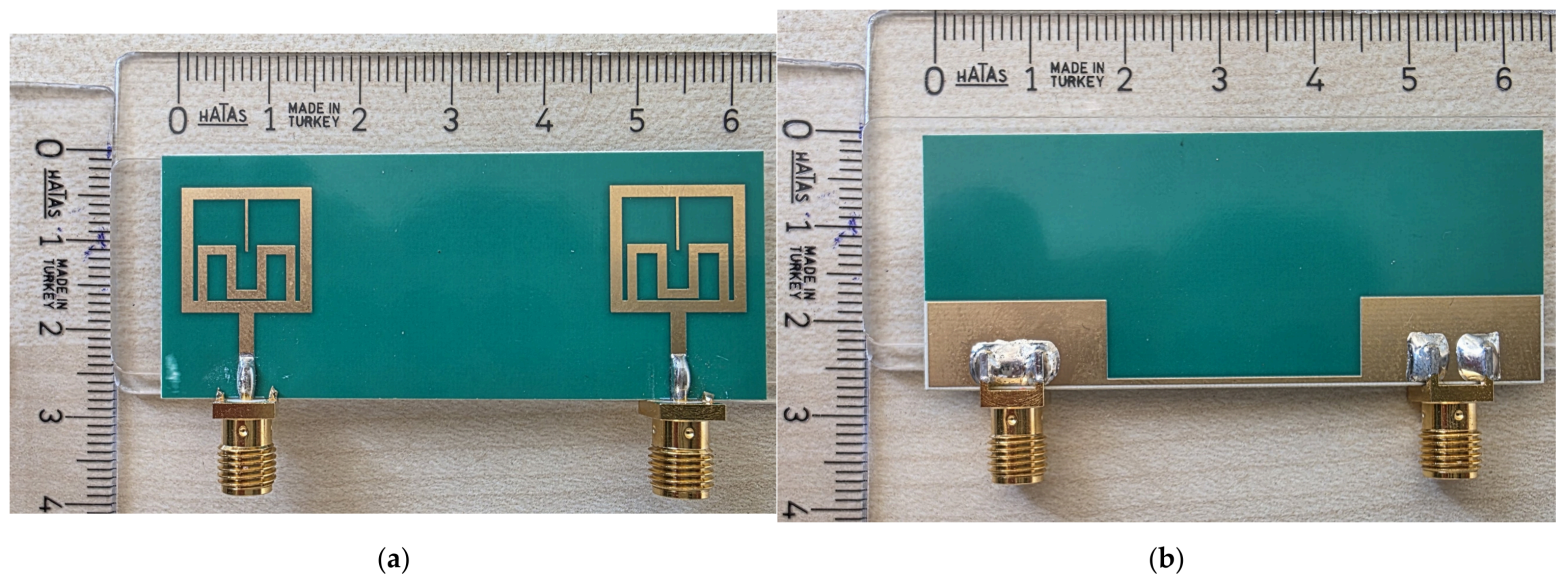
Fabricated prototype of the AI-optimized highly miniaturized dual-band MIMO antenna: (**a**) top view showcasing the nested-loop radiators; (**b**) bottom view displaying the defected ground structure (DGS) and SMA connectors.

To empirically validate the simulated results, the AI-optimized MIMO antenna was fabricated using standard photolithographic etching processes. As shown in Figure 8, the prototype was printed on a 0.762 mm thick Rogers RO4350B high-frequency substrate. Adhering to the specified manufacturing parameters, the 35 µm bare copper traces were directly patterned on the dielectric material without any additional surface plating. For experimental signal excitation, two 50 Ω edge-mount female SMA connectors (Samtec, New Albany, IN, USA, Part No: SMA-J-P-H-ST-EM1) were manually soldered to the microstrip feedlines. The final fabricated prototype maintains an extremely compact overall physical footprint of 25.89 mm × 63.11 mm (1634 mm^2^).

**Figure 9:**
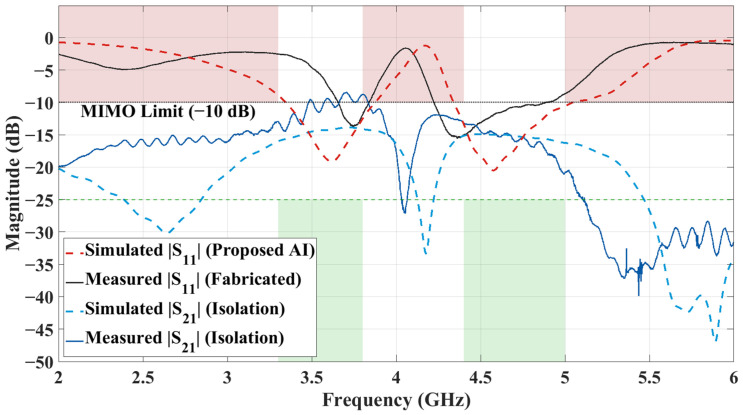
Experimental validation: comparison of the simulated and measured scattering parameters (|S_11_| and |S_21_|). The simulated |S_11_| and |S_21_| responses are shown by the red and light-blue dashed curves, while the measured |S_11_| and |S_21_| responses are shown by the gray and dark-blue solid curves, respectively. The green shaded areas indicate the target operational MIMO passbands, whereas the red shaded areas represent the out-of-band rejection zones.

Following the experimental setup detailed previously, the passive network parameters of the fabricated prototype were extracted and analyzed. Figure 9 presents a comprehensive comparison between the CST-simulated and the measured reflection (|S_11_|) and mutual coupling (|S_21_|) coefficients.

As illustrated, the measured |S_11_| responses (represented by the solid black curve) demonstrate an excellent agreement with the simulated predictions (dashed red curve). The physical antenna successfully captures the dual-band resonance characteristics precisely within the predefined green target regions, achieving deep impedance matching at both the lower and upper sub-6 GHz operating bands. A negligible frequency shift is observed in the measured resonance dips, which is a widely anticipated phenomenon in experimental high-frequency electromagnetics. These minor deviations may arise from a combination of standard manufacturing tolerances, including slight over-etching of the bare copper traces, marginal variations in the substrate relative permittivity (εr), and parasitic effects introduced by the physical SMA connectors and soldered feed regions.

Regarding inter-port coupling, the simulated |S_21_| response remains below −13.8 dB and −14.9 dB across the lower and upper target passbands, respectively. The measured |S_21_| response exhibits frequency-dependent deviations from the simulation and reaches approximately −8.4 dB in the inter-band rejection region.

The measured inter-band |S_21_| discrepancy may result from practical feed and assembly effects that are not fully represented in the idealized simulation, including the finite metallic bodies of the closely spaced SMA connectors, solder joints, and local fabrication variations. As visible in Figure 8b, the connectors and soldered feed regions are positioned close to one another and may introduce additional parasitic coupling. Accordingly, the isolation values of 13.8 dB and 14.9 dB reported in this study refer to the simulated complete two-port model rather than to the measured response. The simulated ECC and CCL results presented in Section 3.2 separately characterize the modeled two-port antenna within the defined operating regions.

### 4.2. Far-Field Radiation Characteristics

**Figure 10:**
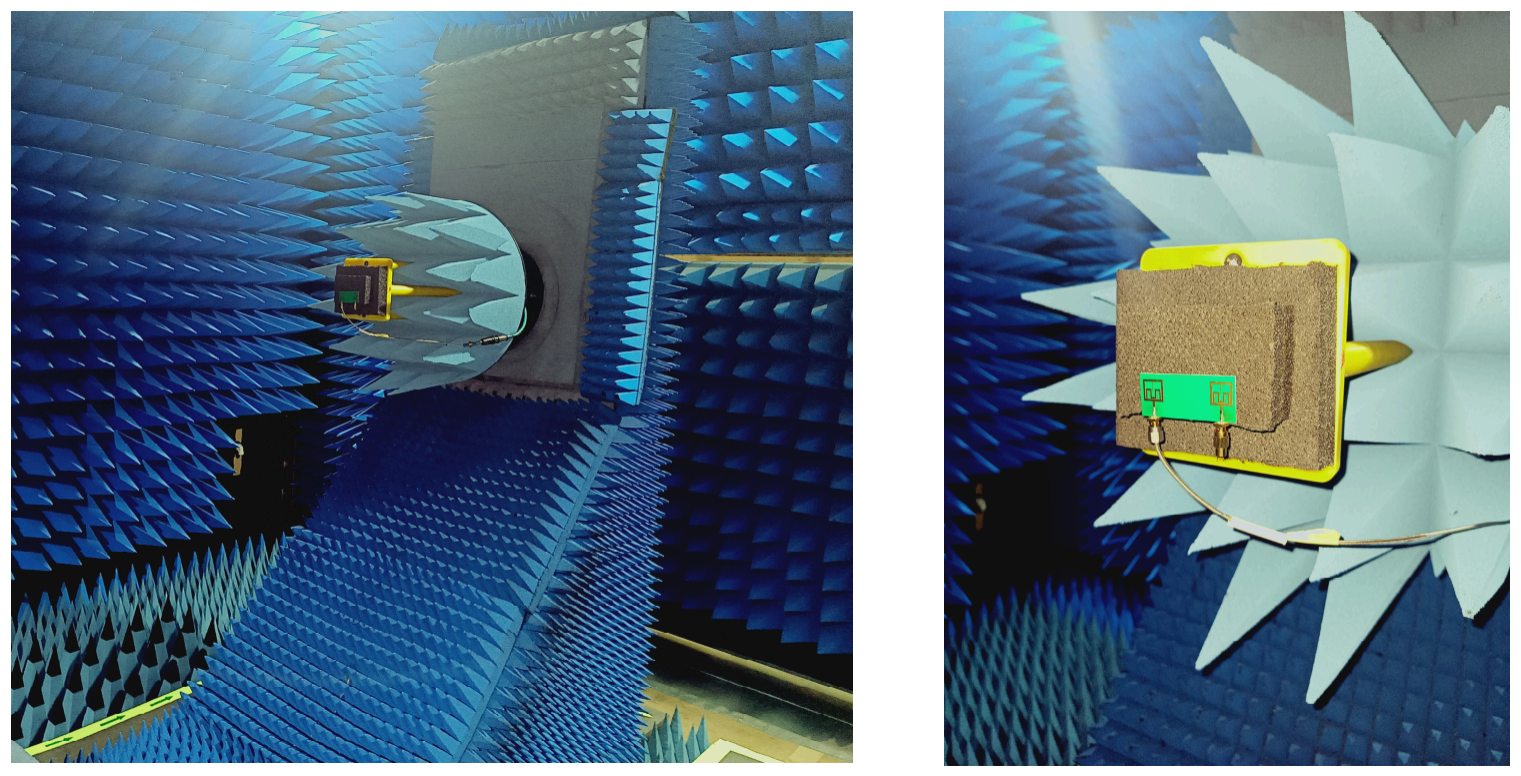
Experimental setup of the fabricated MIMO antenna prototype inside the Spherical Near-Field Measurement System (SNFMS) anechoic chamber.

Following the successful validation of the network parameters, the spatial radiation properties of the AI-optimized MIMO antenna were empirically evaluated to characterize its spatial radiation behavior. Comprehensive far-field measurements were conducted within the fully calibrated Spherical Near-Field Measurement System (SNFMS) anechoic chamber at the TUBITAK BILGEM ATAM facility. As depicted in the experimental setup in Figure 10, the fabricated prototype was firmly mounted on a low-dielectric measurement fixture to minimize background scattering and was evaluated across the full three-dimensional spatial sphere using a standard wideband horn antenna as the probing receiver.

**Figure 11:**
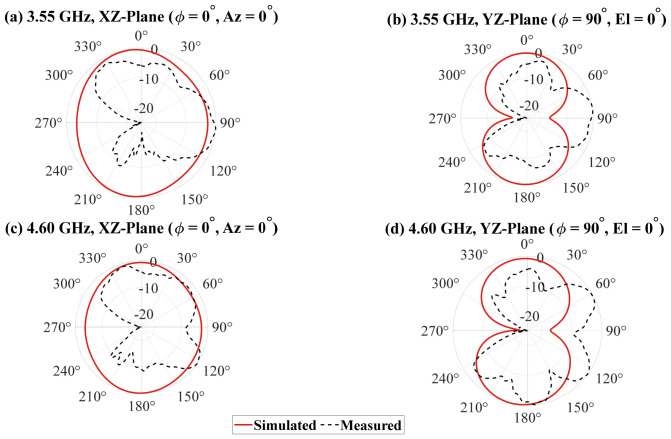
Simulated (solid red) and measured (dashed black) normalized 2D far-field radiation patterns: (**a**) 3.55 GHz in the XZ plane (φ = 0°, Az = 0°); (**b**) 3.55 GHz in the YZ plane (φ = 90°, El = 0°); (**c**) 4.60 GHz in the XZ plane (φ = 0°, Az = 0°); and (**d**) 4.60 GHz in the YZ plane (φ = 90°, El = 0°).

Figure 11 compares the normalized two-dimensional radiation pattern shapes at 3.55 and 4.60 GHz in the principal XZ (φ = 0°) and YZ (φ = 90°) planes. In each panel, the simulated and measured curves were independently normalized to their respective maximum values; therefore, the comparison represents pattern shape rather than absolute gain. Values below −25 dB were clipped only for visual clarity. A fixed 90° angular offset was applied solely to align the simulated and measured coordinate conventions; no smoothing, interpolation, or synthetic modification was applied to the measured data. The simulated responses exhibit a quasi-omnidirectional trend in the XZ plane and bidirectional or sectorial behavior in the YZ plane. The measured responses show partial correspondence with these broad pattern tendencies but exhibit pronounced angular asymmetry and differences in the local lobe and null positions.

The measured and simulated cuts share some broad qualitative pattern features; however, the measured responses exhibit local ripples and differences in the lobe levels and null positions. These deviations may arise from the finite mechanical mounting fixture, SMA connectors and solder joints, RF feed cables, fabrication tolerances, and practical alignment of the simulated and measured coordinate systems. Accordingly, Figure 11 should be interpreted as a comparison of normalized radiation pattern shapes rather than as point-by-point agreement or absolute gain validation. The absolute gain, directivity, and total radiation efficiency are reported separately in Table 3.

Finally, the simulated peak gain and the measured peak gain, directivity, and total radiation efficiency for both ports are summarized in Table 3 to quantify the absolute radiation performance of the antenna. Across both ports, the measured peak gains range from 2.55 to 4.54 dBi at the four evaluated frequencies, with total radiation efficiencies of approximately 51–63%. In the context of applied electromagnetics, extreme physical miniaturization inherently imposes fundamental limitations on radiation resistance. Considering the aggressive physical compactness of the nested loop footprint (merely 1634 mm^2^) and the incorporation of a partial defected ground structure, maintaining a radiation efficiency consistently above 50% constitutes a remarkable operational success. These measured far-field metrics indicate that the fabricated compact radiator maintains practical gain and efficiency levels within the investigated sub-6 GHz operating bands.

The present antenna is linearly polarized, and circular polarization was neither a design objective nor experimentally evaluated in this study. Accordingly, the reported results are most directly applicable to sub-6 GHz links in which antenna orientation and polarization alignment can be reasonably controlled. For IoT scenarios involving arbitrary device orientation, polarization mismatch, or pronounced multipath-induced depolarization, circularly polarized or polarization-diverse implementations may provide greater robustness. Accordingly, the applicability of the present linearly polarized implementation should be interpreted within these orientation and polarization constraints.

## 5. State-of-the-Art Comparison

To objectively evaluate the physical footprint, electromagnetic robustness, and methodological superiority of the proposed AI-optimized MIMO antenna, a comprehensive state-of-the-art comparison is conducted against the recently published (2021–2025) literature. To maintain strict academic rigor and ensure a pristine benchmark, this comparative analysis exclusively prioritizes high-impact, peer-reviewed journal publications indexed in the Science Citation Index Expanded (SCIE) database. The comparative metrics, systematically summarized in Table 4, encompass physical dimensions, substrate material, operating frequencies, inter-port isolation, peak gain, Envelope Correlation Coefficient (ECC), and the deployed optimization methodology. All comparative data points have been rigorously extracted from the explicitly declared experimental and simulation results in the respective original studies.

The consolidated data highlight an engineering trade-off between physical miniaturization, impedance bandwidth, and inter-port isolation. At sub-6 GHz frequencies, minimizing the physical footprint typically incurs severe degradation in operational bandwidth. For instance, Dong et al. [49] reported measured −10 dB impedance bandwidths of 380 MHz (3.34–3.72 GHz) and 560 MHz (4.57–5.13 GHz) within a compact 28 × 28 mm^2^ footprint. Similarly, device-dependent designs like Hu et al. [52] limit their geometric viability solely to specific smartphone chassis configurations, offering no standalone modularity. Alternatively, some designs employ larger reported physical footprints, as illustrated by John et al. [2] (2850 mm^2^), whereas Addepalli et al. [30] reported the antenna dimensions in normalized form as 0.997λ_0_ × 0.997λ_0_ at 3.25 GHz. Although some of the designs summarized in Table 4 provide broader continuous operating bandwidths, the objective of the present work was not to maximize absolute bandwidth alone. Instead, the proposed antenna was developed to balance physical miniaturization, dual-band coverage, inter-port isolation, and planar fabrication simplicity. Within a compact footprint of 1634 mm^2^, the design provides impedance bandwidths of 530 MHz and 710 MHz in the lower and upper bands, respectively, which are wider than the measured bandwidths of 380 MHz and 560 MHz reported in [49]. These results should therefore be interpreted in terms of the combined miniaturization–bandwidth–isolation trade-off rather than as a claim of the widest bandwidth among the compared designs.

The profound impact of dielectric substrate selection and structural complexity on high-frequency radiation capability constitutes another critical divergence in the literature. Recent trends emphasize flexible and optically transparent material platforms for wearable and conformal antenna applications [2,50]. These platforms provide important advantages in terms of mechanical flexibility, conformability, and optical transparency, although their electromagnetic and fabrication characteristics differ from those of rigid low-loss microwave laminates. For example, Desai et al. [50] employed an AgHT-4 transparent conductive layer on a Melinex substrate and reported a maximum gain of 0.53 dBi for their flexible wideband MIMO configuration. Other studies have introduced additional structural mechanisms to enhance gain or inter-port isolation. Hasan et al. [53] utilized a metasurface reflector separated from the MIMO antenna by a 12 mm air gap, whereas Hu et al. [52] employed multiple orthogonal modes in a smartphone-oriented antenna pair. Although these approaches provide application-specific performance benefits, they involve material, structural integration, and device dependence considerations that differ from the planar DGS configuration adopted in the present work.

By employing a planar defected ground structure (DGS) on a Rogers RO4350B laminate with a loss tangent of 0.0037, the proposed design achieves a maximum measured peak gain of 4.54 dBi across the two evaluated ports and simulated inter-port isolation better than 13.8 dB and 14.9 dB across the lower and upper target passbands, respectively, without requiring complex three-dimensional parasitic elements or lossy flexible laminates.

A further distinction of the present study lies in the optimization methodology adopted to obtain the selected antenna geometry. The studies summarized in Table 4 employ a broad range of design strategies, including manual trial-and-error procedures, parametric sweeps, Characteristic Mode Analysis (CMA)-assisted refinement, structure-specific decoupling techniques, and machine learning-based prediction [49,50,51,52,53]. Within these workflows, CMA provides valuable physical insight into modal behavior and surface-current distributions and can guide the subsequent refinement of antenna dimensions, as demonstrated in studies such as Addepalli et al. [30] and John et al. [2]. Narayanaswamy et al. [43] incorporated a Random Forest model into the design process of a four-port antenna operating at 5.90 GHz, illustrating the potential of machine learning to support topology-specific antenna development. In the present study, surrogate prediction, adaptive search space reduction, periodic model updating, Bayesian candidate generation, and repeated CST validation were integrated within a unified closed-loop active learning framework.

Within the predefined campaign, the best CST-validated candidate was identified at the 83rd active learning cycle. This result demonstrates the potential of surrogate-assisted active learning to reduce the number of full-wave evaluations required to identify a competitive dual-band geometry within the investigated design space.

## 6. Conclusions

This article presented the machine learning-assisted optimization and experimental validation of a highly miniaturized dual-band MIMO antenna for sub-6 GHz applications. The proposed Deep Surrogate Active Learning framework combined offline LHS-based data generation, DNN surrogate modeling, and closed-loop active learning with dynamic boundary reduction. The initial surrogate model was trained using 440 valid full-wave responses retained from the offline DOE stage, and the best CST-validated candidate was identified at the 83rd active learning cycle. Under the common maximum search budget of 800 candidate evaluations, the proposed framework provided the most balanced satisfaction of the prescribed lower-band, upper-band, and inter-band target requirements among the evaluated methods.

Structurally, the selected geometry occupies a compact planar footprint of 1634 mm^2^ (25.89 mm × 63.11 mm) on a Rogers RO4350B substrate. Despite the edge-to-edge element spacing of 26.83 mm, the complete two-port model maintained inter-port isolation better than 13.8 dB across the lower target passband and 14.9 dB across the upper target passband. The simulated ECC remained below 0.035 across the target passbands, and the TARC stayed below −10 dB within the effective core MIMO sub-bands of 3.45–3.75 GHz and 4.45–4.90 GHz. Within these core regions, the CCL remained below the 0.4 bps/Hz acceptance threshold and reached minimum values of approximately 0.15 bps/Hz near the resonant frequencies.

Experimental validation comprised VNA measurements of the scattering parameters and spherical near-field measurements of the far-field response in a calibrated anechoic chamber. The fabricated prototype retained the intended dual-band behavior, achieved a maximum measured gain of 4.54 dBi, and exhibited total radiation efficiencies of approximately 51–63% across both ports at the four evaluated frequencies. The measured normalized radiation patterns showed partial qualitative agreement with the simulated cuts, while local deviations were observed under practical feed, mounting, fabrication, and coordinate alignment conditions. Taken together, the measured S-parameter and far-field results support the practical feasibility of the selected compact planar MIMO geometry for the investigated sub-6 GHz operating bands.

The scope of the present validation is limited to the parameterized nested loop, dual-band sub-6 GHz topology investigated in this study. Extending the proposed framework to other antenna architectures or broader frequency ranges would require redefinition of the design variables and retraining with topology-specific full-wave data. Temperature, humidity, thermal cycling, and long-term aging tests were not performed and therefore remain outside the current validation scope. Future studies will address environmental and long-term reliability, bandwidth enhancement strategies that preserve the compact footprint and inter-port isolation, and circularly polarized or polarization-diverse variants for IoT scenarios involving arbitrary device orientation and polarization mismatch.

## Figures and Tables

**Table 1:** Geometrical dimensions and AI-optimized physical parameters of the proposed MIMO antenna.

Parameter Name	Description	Value/Range (mm)
Fixed Dimensions		
substrate_height	Substrate thickness (Rogers RO4350B)	0.762
wd	Edge-to-edge distance between antenna elements	26.83
feed_line_length	Length of the 50 Ω microstrip feedline	9.20
feed_line_width	Width of the 50 Ω microstrip feedline	1.47
groundplane_length	Length of the partial ground plane	9.00
groundplane_width	Width of the individual partial ground section	18.14
gw	Ground plane gap width	0.40
length_1	Length of the meander section of the inner loop	5.57
length_2	Inner distance of the meander section	2.20
height_3	Height of the capacitive tuning stub	5.55
line_width_3	Line width of the capacitive tuning stub	0.015
line_width_1	Trace width of the outer large loop	1.14
line_width_2	Trace width of the inner loop	0.88
AI Search Bounds		
height_1	Optimization limit for outer loop height	[9.88–18.36]
height_2	Optimization limit for inner loop height	[4.38–8.13]
width_1	Optimization limit for outer loop width	[10.45–19.40]
width_2	Optimization limit for inner loop width	[6.86–12.74]
Selected CST-Validated Geometry		
height_1	Selected height of the outer large loop	13.13
height_2	Selected height of the inner loop	5.87
width_1	Selected width of the outer large loop	13.74
width_2	Selected width of the inner loop	8.49

**Table 2:** Benchmarking of the proposed framework and the CST built-in optimizers under a common maximum search budget of 800 full-wave candidate evaluations.

Optimization Method	Evaluation Record	Height_1 (mm)	Height_2 (mm)	Width_1 (mm)	Width_2 (mm)	Final Algorithmic Status
Search Space Bounds	–	[9.88–18.36]	[4.38–8.13]	[10.45–19.40]	[6.86–12.74]	–
Initial (Baseline)	1	11.30	5.00	11.94	7.84	Common reference geometry
NMSA (Nelder-Mead)	144	13.53	6.47	14.42	9.29	Terminated after 144 recorded evaluations; target masks not fully satisfied
TRF (Trust Region)	799	13.26	6.18	14.64	9.25	Best result obtained at Run ID 176; no subsequent improvement observed
GA (Genetic Alg.)	801	15.52	6.29	13.77	10.71	Full budget completed; upper-band matching target not satisfied
Proposed AI Framework	800 attempted; 735 valid responses stored *	13.13	5.87	13.74	8.49	Best CST-validated candidate identified at active-learning cycle 83

* Note: For the proposed framework, the predefined search budget comprised 500 offline LHS trials and 300 online active learning attempts; the baseline geometry was evaluated separately as a common reference. After unsuccessful or interrupted CST runs were excluded, the initial database contained 440 valid full-wave responses, including the baseline response. Of the 300 online attempts, 295 generated valid stored responses, resulting in a final database of 735 entries. The best CST-validated geometry corresponded to Run ID 523 and was identified at the 83rd active learning cycle.

**Table 3:** Simulated peak gain and measured far-field radiation metrics of the fabricated prototype.

Frequency (GHz)	Simulated Peak Gain (dBi)	Measured Peak Gain P1/P2 (dBi)	Measured Directivity P1/P2 (dBi)	Measured Total Efficiency
3.55	~2.90	2.552/3.068	5.383/5.709	−2.830 (~52%)/−2.641 (~54%)
3.76	~3.15	3.517/2.704	6.440/5.393	−2.923 (~51%)/−2.690 (~54%)
4.33	~3.30	3.442/4.537	5.632/6.575	−2.190 (~60%)/−2.037 (~63%)
4.60	~3.25	2.667/4.268	5.455/6.929	−2.788 (~53%)/−2.662 (~54%)

Note: Measured values are reported in Port 1/Port 2 order.

**Table 4:** Performance comparison of the proposed AI-optimized MIMO antenna with the recent state-of-the-art literature.

References	Dimensions (mm^2^ or Normalized λ_0_)	Substrate	Operating Bands (GHz)	Iso. (dB)	Gain (dBi)	ECC	Design/Optimization Method
John et al. [2]	57.00 × 50.00 (2850)	Polyimide	3.60–3.80 & 5.65–5.95	>22	7.70	<0.050	CMA + Parametric Sweep
Addepalli et al. [30]	0.997λ_0_ × 0.997λ_0_ (λ_0_ at 3.25 GHz)	FR-4	3.25–3.90 & 4.37–4.95	>17.5	~4.00	<0.005	Star-fractal slot and DGS-based parametric design
Dong et al. [49]	28.00 × 28.00 (784)	FR-4	3.34–3.72 & 4.57–5.13	>23 (meas.)	2.46	<0.01164	Manual Parametric Sweep
Narayanaswamy et al. [43]	N/R (Tapered)	FR-4	5.90 (Single-Band)	>25	7.85	<0.010	Machine Learning (Random Forest)
Desai et al. [50]	N/R (Flexible)	Melinex (AgHT-4 conductor)	2.21–6.00 (Wide)	>15	0.53	Low	Parametric Sweep
Benghanem et al. [51]	30.00 × 60.00 (1800)	FR-4	2.41–6.00 (Wide)	>18	4.64	<0.008	Parametric Sweep
Hu et al. [52]	N/R (Smartphone)	FR-4	Dual-Band 5G	High	High	Low	Orthogonal Modes/Sweep
Hasan et al. [53]	80 × 80 (metasurface reflector)	Rogers RT5880	3.08–7.75	>15.5	8.5 (meas.)	<0.004	Metasurface reflector
This Work	25.89 × 63.11 (1634)	RO4350B	3.35–3.88 & 4.34–5.05	>13.8/>14.9 (sim.)	4.54	<0.035	Deep Surrogate Active Learning

Note: “N/R” indicates that the explicit footprint dimensions were not reported in the absolute numerical metrics of the cited article. “High/Low/Enhanced” denotes qualitative achievements without precise empirical boundaries.

## Data Availability

The original contributions presented in the study are included in the article; further inquiries can be directed to the corresponding author.
